# Why are there long waits at English emergency departments?

**DOI:** 10.1007/s10198-019-01121-7

**Published:** 2019-10-24

**Authors:** James Gaughan, Panagiotis Kasteridis, Anne Mason, Andrew Street

**Affiliations:** 1 Centre for Health Economics, University of York, York, UK; 2 Department of Health Policy, London School of Economics and Political Science, London, UK

**Keywords:** Emergency department (ED), Accident and emergency (A&E), National health service (NHS), Waiting time, Length of stay, I10

## Abstract

A core performance target for the English National Health Service (NHS) concerns waiting times at Emergency Departments (EDs), with the aim of minimising long waits. We investigate the drivers of long waits. We analyse weekly data for all major EDs in England from April 2011 to March 2016. A Poisson model with ED fixed effects is used to explore the impact on long (> 4 h) waits of variations in demand (population need and patient case-mix) and supply (emergency physicians, introduction of a Minor Injury Unit (MIU), inpatient bed occupancy, delayed discharges and long-term care). We assess overall ED waits and waits on a trolley (gurney) before admission. We also investigate variation in performance among EDs. The rate of long overall waits is higher in EDs serving older patients (4.2%), where a higher proportion of attendees leave without being treated (15.1%), in EDs with a higher death rate (3.3%) and in those located in hospitals with greater bed occupancy (1.5%). These factors are also significantly associated with higher rates of long trolley waits. The introduction of a co-located MIU is significantly and positively associated with long overall waits, but not with trolley waits. There is substantial variation in waits among EDs that cannot be explained by observed demand and supply characteristics. The drivers of long waits are only partially understood but addressing them is likely to require a multi-faceted approach. EDs with high rates of unexplained long waits would repay further investigation to ascertain how they might improve.

## Introduction

Waiting time in emergency departments (EDs) is a major policy concern internationally [1–3]. Long waits can adversely affect patients, in terms of longer inpatient stays [4], higher mortality rates [5, 6], and an increase in the costs of care. In England, a target that 98% of patients should be assessed and admitted, transferred or discharged within 4 h was introduced in 2004 and was initially followed by a significant reduction in long (over 4 h) waits [7, 8]. In 2010, the target was reduced to 95%, but this revised goal has not been met nationally since July 2015 [9].

From 2011/12 to 2015/16, there were on average 1.1 million waits over 4 h each year, breaching the national target by around 400,000.^1^ In part, the pattern of recent breaches of ED waiting time targets reveals mounting pressure on EDs: attendances to English EDs have risen steadily from 21.5 million in 2011/12 to 22.9 million in 2015/16 [10]. Population ageing means this trend is expected to continue as people aged 65 or over (65 +) constitute one-fifth of ED attendances [9] and have higher rates of attendance [11].

In March 2019, NHS England published its plans to overhaul targets across the NHS, including the 4-h waiting time target in EDs [12]. The Powis review of access standards at English EDs underscored continued policy concerns with long waits [13]. The review proposed four new ED standards, including mean waiting time in EDs and a 1-h maximum wait for patients in need of urgent care, to replace the 4-h target. These proposed new standards aim to align incentives faced by hospital managers and clinicians with clinical best practice. It is currently unclear how the proposed measures would be converted into targets for EDs and, hence, to what extent incentives will be aligned. A stronger evidence base for understanding drivers of long waits can assist policy makers in the ongoing process of updating and refining incentives to maximise the quality of care for patients [14].

Commentators have pointed to a range of factors to explain long waits. Higher volumes of non-urgent visits, influenza outbreaks, inadequate staffing and hospital bed shortages can give rise to overcrowding in EDs [15], leading to longer waits for attendees to be assessed, prioritised and treated [6, 15, 16]. With one exception, previous studies have examined the impact of single factors on breaches of the 4-h target, but not explored the effects of these factors in combination [2, 11] or have been limited to a single ED [7]. In a more comprehensive analysis, Keogh et al. explore the relationship between breaches of the 4-h waiting time target and other performance measures, notably cancelled operations, using quarterly data from 2011 to 2016 [17].

We employ a retrospective observational study to examine the effects of a range of factors on all attendances to major EDs in England from 2011/12 to 2015/16. We contribute to the evidence base in four ways. First, we exploit weekly rather than quarterly data, allowing us to identify smaller effects that may still be important from a clinical or policy perspective. Second, we test for effects on two measures of long waits: ‘long overall waits’, which capture the number of waits exceeding 4 h in the ED, and ‘long trolley waits’, which capture the number of patients waiting over 4 h on a trolley (gurney) between the decision to admit and admission to an inpatient ward. As trolley waits affect a subset of attending patients and part of their journey through EDs, they might be affected by different factors to the overall measure. For example, the availability of inpatient beds might be expected to have a more direct impact on the waiting time of patients in need of admission. Third, we investigate a range of demand and supply factors in combination to explain these long waits. Fourth, we explore residual variation in long waits among EDs after accounting for observable demand and supply factors. This allows us to identify EDs with more frequent long waits than expected. These EDs would repay further investigation.

## Data

We use information from all Hospital Trusts with a major ED in England, excluding EDs in specialist children’s hospitals,^2^ from April 2011 to March 2016. A Trust is an administrative unit managing the provision of hospital care and responsible for one or multiple EDs within the local vicinity. Our analysis is by Trust because the data we use to measure attendances and 4-h waits are reported for Trusts and the Trust is the level at which policy makers generally interact with providers. A major ED is open 24 h a day on all days of the year, has full resuscitation facilities and is led by a senior physician, referred to in England as a ‘hospital consultant’. Major EDs are the primary providers of emergency medicine in England, accounting for 79% of ED attendances in 2015/16, excluding attendances to specialist children’s hospitals.^3^

### Outcome measures

We consider two measures of long (over 4 h) waits: long overall waits and long trolley waits. Counts of these events are constructed from the A&E^4^ Attendances and Emergency Admissions statistical collection, published by NHS England and analysed at the weekly level [18].^5^ The first measure, ‘long overall waits’, is the number of attendances to the ED in a week that last more than 4 h, from arrival to the patient being dealt with. Here, ‘dealt with’ covers discharge alive or dead, inpatient admission or transfer elsewhere [19]. The second measure comprises the number of ED attendances lasting more than 4 h after a decision has been made to admit the patient as an inpatient. We refer to this as ‘long trolley waits’, because it reflects the time spent waiting on a trolley (sometimes in the ED or a hospital corridor) before an appropriate inpatient bed becomes available. For example, a patient might arrive at A&E, wait 3 h for initial assessment, at the end of which a decision is made to admit them. They might wait a further 2 h to be admitted. In this case, the overall wait would be 5 h and the trolley wait would be 2 h.

As a patient must attend an ED to experience a long wait and because EDs differ in the number of attendances they receive, we include an exposure term of number of ED attendances. That is, the logarithm of the number of ED attendances is included as a variable with a coefficient constrained to 1. This means the models explain variation in rates of long waits (numerator) over attendances (denominator).

### Explanatory variables

We include three groups of explanatory variables: (1) demand variables capturing features of the local population and ED attendees; (2) supply variables in terms of characteristics of the ED, the hospital in which the ED is based, and characteristics of long-term care (LTC) supply in the local area; and (3) time factors (years, and months of the year to capture seasonal effects).

#### Demand variables

We include attendee demographic and clinical characteristics of age, gender and number of diagnoses.^6^ These characteristics are taken from the Hospital Episode Statistics (HES), a patient-level dataset of all ED attendances to NHS Trusts. [20] We employ variables that capture the weekly average characteristics of attendees to each ED.^7^

We also include the percentage of attendees who live in the lowest quintile of income deprivation, assessed using the English index of multiple deprivation 2010 [21]. Jones and Wildman [22] found patients with lower income had poorer self-assessed health. Such patients may take longer to assess as a consequence.

We include three mutually exclusive methods of leaving EDs, namely the percentage of attendees who leave an ED without being treated; the percentage admitted to an inpatient ward; and the death rate per 1000 ED attendances.

Finally, we include the percentage of people aged 65 + living within 10 km, to capture demand of the local population. People aged 65 + are more frequent users of ED services [11].

#### Supply variables

We include variables to measure characteristics of the ED or Trust in which it is based. These factors are, in principle, more likely to be under the control of the ED than demand side factors. We capture staffing arrangements as the ratio of full time equivalent (FTE) ED physicians over number of attendances. The numerator is from the Electronic Staff Record (ESR), a census taken at the end of each quarter in 2011/12 and each month from April 2012 [23].^8^ The denominator is a count of weekly attendances, in thousands. All else being equal, we would expect a higher ratio of physicians to attendances would mean patients are seen more quickly, thereby reducing the frequency of long waits.

We also include a dummy variable taking the value 1 if the ED is in a Trust that also has a Minor Injury Unit (MIU), Urgent Treatment Centre or Walk-in Centre and 0 otherwise [24].^9^ These units, hereafter jointly referred to as MIUs, were created to relieve pressure on EDs by caring for less severely ill patients. We would, therefore, expect fewer long waits in EDs with co-located MIUs.

We account for Trust inpatient bed occupancy rates, published quarterly by NHS England [25]. For each quarter, we calculate the average percentage of available overnight beds that are occupied, after excluding maternity beds.^10^ The higher the occupancy rate, the lower the capacity to admit new patients from EDs [17, 26, 27]. This might result directly in a wait over 4 h for patients to be admitted, or indirectly affect all ED patients by contributing to crowding within the ED.

We account for delayed transfers of care (DTOCs), which are drawn from monthly situation reports published by NHS England [28]. We use the percentage of available bed-days ‘lost’ in each month to capture discharge delays from a hospital.^11^ A higher percentage may be associated with more long waits because a delayed discharge from hospital reduces bed availability for patients waiting to be admitted from an ED. Inpatient delays may also capture unobserved aspects of the quality of patient flows, perhaps due to internal bed management arrangements or to the availability of post-discharge care.

We also account for the local supply of long-term care (LTC), captured as the rate of care home^12^ beds per head of population aged 65 + in the local area. A higher rate of care home beds may reduce delayed transfers out of hospital and therefore reduce hospital bed occupancy. To calculate the rate, we use as a numerator the number of registered care home beds for people aged 65 + (including beds for people with dementia) within a 10-km radius of the ED,^13^ sourced from monthly snapshots published by the sector regulator, the care quality commission (CQC) [29].

#### Time trends and seasonality

The demand for and supply of ED care, hospital and long-term care are all subject to temporal variation. A set of dummy variables for month of the year represents cyclical patterns of need, most notably the influenza season during winter months. We also include a set of year dummy variables, which capture general changes in need over time as well as national policy changes that might affect waiting time.

## Methods

We use a count model to investigate variation across EDs and over time in the number of long overall waits and long trolley waits. This approach explicitly recognises the distributional characteristics of a dependent variable with integer values. A Poisson model with ED fixed effects (FEs) [30, 31] is employed for this analysis. The conditional mean of the model is presented in Eq. 1

1 $$\lambda_{it} = \exp (D_{it} \beta_{1} + S_{it} \beta_{2} + T_{t} \beta_{3} + \log ({\text{ATT}}_{it} ) + \delta_{i} ),$$

where $\lambda_{it}$ is the conditional expected number of long waits in ED *i* and week *t*, $D_{it}$ and $S_{it}$ are vectors of demand and supply variables as described in the Data section and $T_{t}$ is the set of dummy variables for months and years, where April and 2011 are the base month and year, respectively. ${\text{ATT}}_{it}$ is the number of attendances to the ED. This variable enters the model as an exposure term, and is given a constrained value of 1. The ED-specific FEs $\delta_{i}$ capture unobserved time-invariant effects. We calculate standard errors clustered at the ED level.

We preferred the Poisson fixed effects model for several reasons. First, unlike random effects models, it allows for the unobserved time-invariant variables to be correlated with the observed variables and therefore it provides complete adjustment for these potential correlations.

Second, a well-known limitation of the cross-sectional Poisson model is that it specifies the conditional variance to be equal to the conditional mean (equidispersion). In many applications, the dependent variable is overdispersed, even after conditioning on covariates to allow the Poisson rate to vary across units. This leads to deflated standard errors and low *P* values. By including department fixed effects in the Poisson model, we account for time-invariant unobserved heterogeneity. This is expected to reduce the conditional variance. If there is unobserved heterogeneity that is specific to particular points in time, overdispersion may still exist. For this reason, we calculate standard errors clustered at the ED level.

Third, in the panel data context, the Poisson fixed effects model is more robust than the Negative Binomial fixed effects model. Estimating the NB fixed effects model with unconditional maximum likelihood is subject to potential incidental parameter problems. Estimating by conditional maximum likelihood is feasible with a specific parameterisation of the model [30] but it has been shown that this is not a true fixed effects model controlling for all time-invariant covariates [32].

Fourth, when investigating variation in hospital-specific effects, detailed below, employing fixed effects places no restrictions on the values which can be taken for each Trust level effect, which is not the case if random effects are applied.

Employing fixed effects does not in itself account for serial correlation. However, the implications of serial correlation are limited to inference and we adjust for potential underestimation of standard errors by employing robust standard errors clustered at the department level.

We also present an alternative specification where we consider attendances to Minor Injury Units as well as to EDs. The dependent variable, exposure term and patient characteristics are calculated from the set of attendances to an ED (as in our main specification), plus those attending an MIU. In this way, we account for a wider set of patients receiving treatment, including those with more minor conditions. However, this is not our preferred specification as there is a risk of double counting patients who attend an MIU and then are sent to a co-located ED, or vice versa. These cases cannot be identified from the aggregated data on waits, from which our dependent variables are calculated.

To undertake comparisons across EDs, we derive the indirectly risk-standardised waits ratio (SWR). The SWR is a ratio of observed long waits over the counterfactual expected number of long waits had the given ED been at a hypothetical benchmark. The benchmark is defined as the ED with the average estimated FE. The denominator of the SWR is predicted from the Poisson model using the demand and supply characteristics of the ED and the FE of the hypothetical benchmark ED. Therefore, the SWR captures variation in long waits attributable to the ED-specific FE, not to the demand and supply factors accounted for in the model. An SWR above 1 implies that the ED has a higher rate of long waits than expected, given the demand and supply factors it faces, and might, therefore, repay further investigation to identify ED-specific explanatory characteristics that cannot be observed in routine administrative data.

## Results

### Descriptive statistics

Table 1 presents descriptive statistics for long overall and long trolley waits and the various explanatory variables. Information for two samples is presented: (i) Attendances to Emergency Departments; (ii) Attendances to Emergency Departments and Minor Injury Units. Our analysis includes 139 departments^14^ followed over 260 weeks. Of attendances to EDs alone, around 8% (155/1941) of weekly attendances to the average ED lasted more than 4-h, increasing from 5% in April 2011 to 19% in March 2016. Over the same period, the percentage of long trolley waits increased from 16 to 21% of long overall waits.^15^

**Table 1:** Descriptive statistics

Variable	Period	ED attendances				ED and MIU attendances			
		Mean	SD	Min	Max	Mean	SD	Min	Max
Outcome variables									
Count patients waiting 4 + h	Weekly	155.4	154.0	0	1558	157.0	156.9	0	1609
Count patients waiting 4 + h after decision to admit	Weekly	30.64	47.48	0	554	30.64	47.48	0	554
Exposure term									
Number of attendances	Weekly	1941	850	614	7122	2330	1140	614	9595
Demand variables									
Mean patient age	Weekly	40.60	3.637	21	58.20	40.05	3.519	21	53.98
Mean % males	Weekly	50.35	1.953	30.99	100	50.32	1.961	29.27	100
Mean % most deprived quintile	Single snapshot	22.91	16.46	0	84.20	22.76	16.33	0	84.20
Mean total diagnoses	Weekly	0.839	0.492	0	3.928	0.833	0.486	0	3.928
Mean % untreated	Weekly	3.477	1.906	0	32.36	3.317	1.855	0	32.36
% of ED attendances ending in admission	Weekly	27.06	6.453	0	95.27	27.06	6.453	0	95.27
Mean deaths per 1000 attendances	Weekly	1.490	1.153	0	13.89	1.356	1.064	0	11.92
% of Pop aged 65 + within 10 km	Yearly	16.55	3.702	8.443	26.93	16.55	3.702	8.443	26.93
Supply variables									
Number of physicians per 1000 attendances	Quarterly/monthly*	20.51	6.785	4.504	76.71	17.50	5.752	3.392	66.10
Minor injury unit in Trust	Monthly	0.50	0.500	0	1	0.499	0.500	0	1
% Occupied beds in Trust	Quarterly	86.36	6.219	56.56	99.80	86.36	6.219	56.56	99.80
% of bed-days lost due to delays	Monthly	2.919	2.177	0	17.86	2.919	2.177	0	17.86
Rate care home beds per 1000 Pop aged 65 +	Monthly	44.93	10.43	23.82	91.81	44.93	10.43	23.82	91.81

Observations = 36,057 (260 weeks with on average 139 departments), Study period 2011/12–2015/16

*SD* standard deviation

*Number of FTE emergency physicians is reported quarterly for 2011/12, then monthly from April 2012

**Fig. 1:**
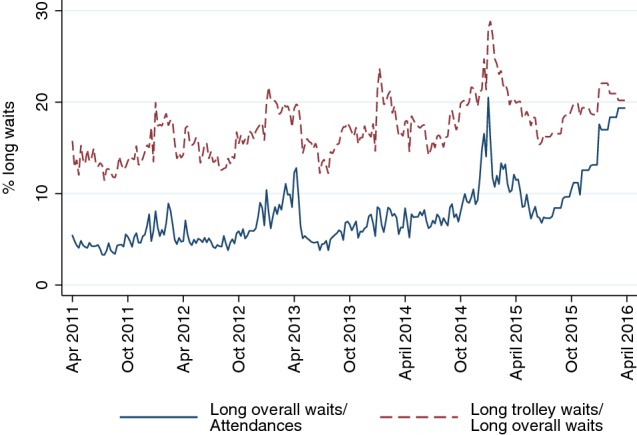
Mean % of weekly waits for ED attendees

Figure 1 presents a line plot of the weekly average rate of long overall waits/ED attendances and long trolley waits/long overall waits. The figure shows these rates have increased over time but there are large week-by-week and seasonal variations that dominate this general trend.

Descriptive statistics indicate that patients attending EDs are more likely to live in more deprived areas, with 23% of patients living in the lowest quintile of deprivation. On average, less than one diagnosis is recorded for each patient, likely to reflect limited diagnostic activity in the ED. Of attendees, 3% leave untreated, 27% are admitted to a hospital ward and 0.15% die while in the ED.

Around 17% of people living within 10 km of an ED are aged 65 +, slightly lower than the national rate. This suggests some relative concentration of younger populations around hospitals, which are more likely to be based in urban areas. Inpatient bed occupancy averages 86%, just above the recommended rate of 85% [2].

Descriptive statistics for the combined sample of attendances to EDs and MIUs are very similar to that of attendances to EDs alone because the vast majority of attendances are to EDs. Information for the combined sample indicates attendances to MIUs are slightly less complex, with a slightly lower average age and lower rate of death. This is to be expected as MIUs were introduced to provide treatment for a less severely ill population. The variable ‘% of ED attendances ending in admission’ is identical in the two samples because it is not possible to calculate this variable separately for ED and MIU attendances. The other variables reporting identical information for the two samples relate to wider populations or services such as inpatient care or long-term care, calculated from the wider Trust or geographical area (see the Data section for details).

### Regression results

In the regression analysis, we model the count of overall or trolley waits lasting more than 4 h (long waits) and report incident rate ratios (IRRs). Results are presented in Table 2.

**Table 2:** Regression results

	Overall waits > 4 h (ED)		Trolley waits > 4 h (ED)		Overall waits > 4 h (ED + MIU)	
(1)	(2)	(3)	(4)	(5)	(6)	(7)
Variable	IRR	ci95	IRR	ci95	IRR	ci95
Mean patient age	1.0416***	1.0224,1.0612	1.0816***	1.0563,1.1075	1.0298***	1.0165,1.0434
Mean % males	0.9868***	0.9796,0.9941	0.9928	0.9831,1.0026	0.9802***	0.9709,0.9896
Mean % most deprived quintile	0.9957	0.9846,1.0069	1.0084	0.9895,1.0276	0.9866**	0.9779,0.9953
Mean total diagnoses	0.9638	0.8953,1.0375	1.0953	0.9592,1.2506	0.9511	0.8800,1.0281
Mean % untreated	1.1508***	1.1151,1.1877	1.0946***	1.0443,1.1472	1.1563***	1.1153,1.1988
% of ED attendances ending in admission	0.9996	0.9914,1.0079	0.9953	0.9816,1.0091	1.0026	0.9941,1.0112
Mean deaths per 1000 attendances	1.0331***	1.0252,1.0411	1.0416***	1.0206,1.0631	1.0371***	1.0279,1.0464
% of Pop aged 65 + within 10 km	0.9872	0.8675,1.1233	1.1729	0.8898,1.5461	1.0056	0.8834,1.1447
Number of physicians per 1000 attendances	1.0017	0.9966,1.0068	0.9978	0.9806,1.0152	0.9923*	0.9854,0.9994
Minor injury unit in Trust	1.1985***	1.1051,1.2997	1.1632	0.9291,1.4564	1.2324***	1.1318,1.3420
% Occupied beds in Trust	1.0145***	1.0092,1.0199	1.0339***	1.0201,1.0478	1.0140***	1.0088,1.0193
% of bed-days lost due to delays	1.0059	0.9870,1.0252	0.9958	0.9620,1.0308	1.0061	0.9871,1.0254
Rate Care Home Beds per 1000 Pop aged 65 +	1.0039	0.9929,1.0151	1.0151	0.9879,1.0430	1.0061	0.9953,1.0171
May	0.8383***	0.8159,0.8612	0.7641***	0.7252,0.8051	0.8303***	0.8080,0.8532
June	0.7815***	0.7576,0.8062	0.6815***	0.6378,0.7283	0.7704***	0.7464,0.7953
July	0.7353***	0.7087,0.7628	0.6217***	0.5841,0.6618	0.7254***	0.6988,0.7530
August	0.7799***	0.7447,0.8167	0.6447***	0.5922,0.7019	0.7931***	0.7583,0.8294
September	0.9299***	0.8921,0.9694	0.9122*	0.8499,0.9791	0.9314***	0.8935,0.9710
October	1.0098	0.9733,1.0477	1.0112	0.9477,1.0789	1.0008	0.9642,1.0387
November	1.1007***	1.0567,1.1464	1.1316***	1.0567,1.2118	1.0877***	1.0440,1.1332
December	1.2090***	1.1631,1.2568	1.2172***	1.1354,1.3049	1.2128***	1.1649,1.2628
January	1.3163***	1.2544,1.3814	1.5095***	1.3965,1.6315	1.3357***	1.2765,1.3976
February	1.3780***	1.3293,1.4284	1.5166***	1.4114,1.6296	1.3831***	1.3327,1.4353
March	1.2956***	1.2467,1.3464	1.3427***	1.2497,1.4426	1.2779***	1.2307,1.3268
2012/13	1.1863***	1.0828,1.2997	1.1605	0.9788,1.3759	1.1805***	1.0764,1.2945
2013/14	1.3116***	1.1586,1.4847	1.2735*	1.0165,1.5955	1.3060***	1.1534,1.4788
2014/15	1.8304***	1.5585,2.1497	1.9832***	1.5255,2.5783	1.8373***	1.5688,2.1518
2015/16	2.1931***	1.8152,2.6498	2.2838***	1.7054,3.0584	2.1900***	1.8204,2.6347
Observations	36,057		35,349		36,057	
AIC	1,228,414		676,310		1,244,317	
BIC	1,228,652		676,548		1,244,555	
s.e.	Robust		Robust		Robust	

*IRR* incidence rate ratio, *ci95* 95% confidence interval, *Robust* department cluster robust standard errors, *Study period* 2011/12–2015/16

Trolley waits begin after a decision has been made to admit a patient. April is the reference month, 2011/12 is the reference year. The coefficient of the exposure term, number of patients attending a major ED in week t, is constrained to take a value of 1

*P < 0.05, **P < 0.01, ***P < 0.001

Results for the analysis of long overall waits are reported in columns 2 (IRR) and 3 (ci95) of Table 2. For the demand variables, we find that an increase in mean patient age by 1 year is associated with a 4.2% increase in the rate of long overall waits, a 1 percentage point increase in male patients is associated with reductions in long overall waits by 1.3%, a 1% increase in the percentage of patients leaving without being treated (‘untreated’) is associated with an increase in the rate of long overall waits of 15.1%, and an increase in the death rate by one in 1000 ED attendances is associated with an increase in long overall waits by 3.3%.

In terms of supply variables, the introduction of an MIU and an increase by 1 percentage point in the inpatient bed occupancy rate are associated with increases in the rate of long overall waits by 19.9% and 1.5%, respectively.

We also find seasonal and annual patterns, with 10 months significantly different from the base month (April). The rate of long overall waits is higher in winter months and lower during the summer. We also observe a significant increase in the rate of long waits over time, captured by year dummy variables.

In general, long trolley waits are affected by similar factors to long overall waits (as reported in Table 2, columns 4 and 5) except that gender has no significant effect on the rate of long trolley waits, nor does the presence of an MIU. The rate of trolley waits is also increasing over time, but is not significantly different from the 2011 base year until 2013/14, instead of 2012/13 for overall waits.

In columns 6 and 7 of Table 2, we report results after replacing the dependent variable, exposure term and patient characteristics from EDs alone with measures that combine ED and MIU attendances. In general, the qualitative findings of this analysis are the same as for overall long waits among attendances to EDs alone. However, we find that a 1 percentage point higher concentration of people in the most deprived quintile is associated with a 1.3% lower rate of long overall waits and an increase of one in the rate of physicians per 1000 attendances is associated with a reduction in long overall waits by 0.8%.

#### Variation across EDs

**Fig. 2:**
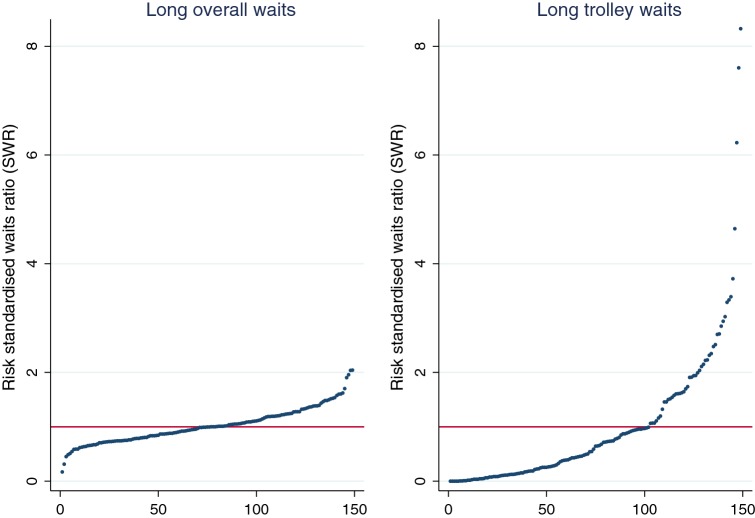
Risk-standardised waits ratios for long overall and trolley waits

In Fig. 2 we plot the SWRs for long overall and long trolley waits separately. The correlation between the two sets of SWRs is quite low (*r *= 0.34), implying that the two wait measures provide different insights into the nature of ED performance. Moreover, for both measures, there is considerable variation in performance: in some EDs the number of long waits is half the predicted level, in others it is twice what would be expected based on the demand and supply characteristics we have accounted for. These differences could be related to differential staffing arrangements or management practice between EDs. To improve performance, attention might be directed at the small cluster of EDs at the right-hand tail of the distribution, with more frequent long overall waits than expected (Fig. 2a) and at the long tail of EDs in which patients experience many more long trolley waits than predicted (Fig. 2b).

## Discussion

We investigate the determinants of long overall waits and trolley waits by exploiting variation over time and across EDs within the English NHS.

The rate of long overall waits is higher in EDs serving older patients and/or those with a higher proportion of females, and in EDs where higher proportions of attendees die or leave before receiving treatment. Long overall waits are also higher at EDs in Trusts with greater bed occupancy or where a co-located MIU is introduced. With the exception of gender and the introduction of an MIU, the same factors are significantly associated with long trolley waits.

As has previously been found [17], our study confirms that long waits are less common in Trusts with lower bed occupancy rates, so efforts to reduce bed occupancy might also help reduce long ED waits by allowing patients to be admitted more quickly to an inpatient bed. Depending on the drivers of inpatient occupancy rates, this might be achieved through an increase in the supply of inpatient beds or by changes to admission and discharge policies.

Both older age and the death of the attendee may be markers of greater case-mix complexity, which might increase waits if complexity prolongs treatment time or demands a more intensive use of resources. The relationship may operate in two directions. In one way, these markers of complexity might lead to longer treatment times for these patients and knock-on delays in care for other ED patients. In the other, people facing longer ED waits may be at greater risk of death, as highlighted in other studies [5, 6].

When more attendances end without treatment, long waits are more frequent. This might reflect a range of scenarios including patients leaving untreated if their expected waiting time is long [19]. EDs also act as a safety net for people suffering a mental health crisis: poor access to specialist mental health services can cause delays [33], and these individuals are more likely to leave EDs without being seen [34]. If this mechanism is the primary one, untreated attendances might be a proxy for additional pressure placed on EDs when mental health services are not available.

Trusts that established MIUs were those with ED waiting time problems. EDs in Trusts with an MIU typically treat a more complex case-mix on average, because less complex patients are directed to the MIU. This probably explains the significant positive association between the introduction of an MIU and long waits.

Previous evidence is mixed regarding the impact of staffing levels on ED waiting times [35, 36] and our study shows that the effect of the ratio of physicians to attendances is statistically insignificant in our main analyses with a small significant negative effect only in our analysis of combined attendances to ED and MIU. Possible reasons for the lack of significance in the main analysis include: (i) The rate of physicians per attendance at EDs might be close to optimal given the production function of each ED and levels of other inputs; (ii) The data on physicians, which are limited to monthly or quarterly snapshots of employees, do not sufficiently capture fluctuations in the ED at shorter intervals. That said, in the analysis of combined ED and MIU attendances, we found that a higher rate of physicians to attendances is associated with fewer long waits, which would accord with expectations.

In all our specifications, neither the percentage of bed days lost due to delays in discharging patients nor the rate of care home beds per 1000 persons have a significant impact on waits. As with data on ED physicians, these data are limited to capturing monthly or quarterly snapshots. The lack of significance may also reflect limited variation in these data, which may arise if the local supply of long-term care is largely determined by need assessment.

Compared to the separate analysis of ED attendances, the combined analysis of ED and MIU attendances found that rates of long waits are lower in areas where levels of deprivation are higher. This might reflect hospital attendances at a lower threshold as a substitute for other forms of care such as GPs in more deprived areas. As less complex patients are treated in MIUs and might be dealt with more promptly in that setting than an ED, the strength of such an effect might be more strongly observed when MIU attendances are included.

While there is wide variation across EDs in their rates of long overall and trolley waits, our analysis detects only a handful of factors that significantly explain this variation. Some of these factors are the ones over which neither EDs nor their host Trusts have any control, in particular the demographic characteristics of patients attending the service.

Although we have not identified a predominant factor that explains why long waits are more frequent in some EDs than others, our analysis is able to identify those EDs with more frequent long overall and trolley waits that cannot be explained by a range of observable demand and supply features. There are several potential mechanisms which might lead to this observation. For example, some EDs might face higher variation in the number of attendances within a week, including more frequent spikes in demand. Alternatively, the variation might reflect differences in policies of assessment, admission and disposal across hospitals. The EDs at the right side of each SWR distribution would repay closer study to ascertain how they might improve and to determine what information is needed to apply a waiting time target as a fair measure of performance.

This work has five main limitations that could be addressed by future research. First, the scope of the study is restricted to factors that affect ED waits conditional upon attendance. We therefore do not consider factors that drive attendances, although these could also influence waits. That said, we allow for staffing and organisational features over which the ED may have the greatest control.

Second, our unit of analysis is the Hospital Trust. Some Trusts have more than one major ED (each located on a separate site within the Trust), so considering multiple departments as a single organisation restricts our ability to control for site-specific staffing or locational characteristics and constraints within Trusts. In addition, whilst we control for average patient characteristics within a Trust, whether an attendee experiences a long wait is likely to depend partly on their individual patient characteristics. As datasets improve in their coverage and granularity [37], future research should consider multilevel modelling to take account of the clustering of patients within EDs, and the presence of multiple EDs within Trusts.

Third, our unit of time is aggregated to the weekly level. Spikes in pressure on EDs can occur over a few hours [36], due to a specific event like a road traffic accident. Our weekly measure is not sensitive to the timing of such spikes in demand but captures persistent high demand from week to week. We are also unable to identify features of weekly attendances which require information at shorter intervals, such as variance in attendances within the week. Daily datasets currently cover only the winter months, which are not a representative portion of a year, but if these are extended to the whole year, it might be possible to gain insights into day-to-day variations in activity and performance [38].

Fourth, we measure staffing levels by the number of physicians in an ED. Waiting times could also be influenced by the availability of other staff, notably ED nurses [35], but these data are not recorded routinely in England.

Finally, there might be additional constraints in the process of assessing and providing care for patients in an ED, leading to longer waits. This could include the level of crowding already present in an ED, though this is partially captured by the number of attendances. There might also be constraints in the speed with which tests can be carried out, due to the availability of equipment needed to perform tests or of the technical staff required to prepare and analyse them. Unfortunately, there are no national data that capture these processes, but could form part of the review of those EDs at the tail of the SWR distribution.

## Conclusion

There is substantial unexplained variation across EDs, with the rate of long waits at a handful of EDs being substantially higher than predicted by the demand and supply characteristics of the ED. The reasons for these differences may not be observable from currently available routine data but insights might be gained by further investigation of those EDs with a high rate of unexplained long waits, to ascertain why their performance is worse than expected.

## Footnotes

1 As the target for all EDs in this period permitted 5% of waits to exceed 4 h, and there were around 14 million stays in each year, around 700,000 long waits might be observed even if every ED met this target.

2 Specialist children’s hospitals have a major ED, referred to as ‘type 1’ in NHS documentation, but provide specialist paediatric care. We limit our analysis to general acute hospitals.

3 Our study also excludes other types of specialist hospitals. These are limited to a single clinical department. In NHS documentation, the EDs of specialist hospitals, which are not children’s hospitals, are referred to as ‘type 2’.

4 A&E, standing for Accident and Emergency, is the term used within the NHS for Emergency Departments.

5 ED Attendances and Emergency Admissions data were published at the weekly level (to June 2015) then at the monthly level (from July 2015). In imputing weekly figures from monthly data, we assume attendances and emergency admissions are uniformly distributed over the month, allocating counts in integers to each week depending on the number of its days in a specific month. For example, a week with 3 days in July and 4 in August, would receive 3/31 of counts from July and 4/31 of counts from August.

6 Diagnosis codes are specific to ED attendances and taken from a list produced by NHS Digital as part of a wider set of commissioning data sets.

7 While waiting times could be constructed from HES, we use official Attendances and Emergency Admissions data to calculate outcome variables as described in the ‘Outcome measures’ section above. These figures are preferred because they are the measure by which the 4-h target is judged by policy makers.

8 When using quarterly or monthly data, we assume that the data are stable over the weeks covered. We allocate weeks to quarters on the basis that there are 13 weeks in each quarter. Each week is allocated to a month according to the month with the majority of days in that week.

9 We identify MIUs in Trusts if there is any activity at a relevant unit, referred to as type 3 A&E, reported by a Hospital Trust in a given month.

10 Patients using a maternity bed generally do not pass through EDs.

11 The denominator is the average number of available overnight beds in each quarter multiplied by the days in the month. This reflects the assumption that the number of available beds is stable over days in a quarter and that the numerator captures delays in a month.

12 Care home beds encompass beds in skilled nursing homes and residential care without nursing.

13 The postcode of the headquarters of the Hospital Trust proxies for the geographical location of EDs.

14 This is the average figure over the entire period. There is some variation in number of departments across weeks due to mergers of Trusts over the period considered.

15 By definition, a long trolley wait is also a long overall wait. The measure of long trolley wait is only reported as an aggregated figure for all ED types, not separately for major EDs. However, around 99% of attendances lasting over four hours are in major EDs. We therefore use attendances to a major ED as the exposure term to avoid potential double counting.
